# A survey of computational tools for downstream analysis of proteomic and other omic datasets

**DOI:** 10.1186/s40246-015-0050-2

**Published:** 2015-10-28

**Authors:** Anis Karimpour-Fard, L. Elaine Epperson, Lawrence E. Hunter

**Affiliations:** Department of Pharmacology, University of Colorado School of Medicine, Aurora, CO 80045 USA; Integrated Center for Genes, Environment, and Health, National Jewish Health, Denver, CO 80206 USA

**Keywords:** Proteomics, Machine learning, Random forests, PLS, PCA, SVM, Proteomics repository

## Abstract

Proteomics is an expanding area of research into biological systems with significance for biomedical and therapeutic applications ranging from understanding the molecular basis of diseases to testing new treatments, studying the toxicity of drugs, or biotechnological improvements in agriculture. Progress in proteomic technologies and growing interest has resulted in rapid accumulation of proteomic data, and consequently, a great number of tools have become available. In this paper, we review the well-known and ready-to-use tools for classification, clustering and validation, interpretation, and generation of biological information from experimental data. We suggest some rules of thumb for the reader on choosing the best suitable learning method for a particular dataset and conclude with pathway and functional analysis and then provide information about submitting final results to a repository.

## Introduction

Proteomics, the assessment and quantitation of protein expression changes in a given type of biological sample, contributes heavily to current views in modern biology, genetics, biochemistry, and environmental sciences. Expression proteomics studies investigate the presence or absence patterns of proteins in disease compared to normal using a mass spectrometry approach often preceded by gel separation methods. Proteomics is a science that focuses on the study of proteins: their roles, their structures, their localization, their interactions, and other factors. Proteomics has emerged as a powerful tool in many different fields and is a technique widely used across biology, mainly applied in disease [1–3], agriculture, and food microbiology. Proteomics is becoming increasingly important for the study of many different aspects of plant functions. For example, it is used to help identify candidate proteins involved in the defensive response of plants to herbivorous insects [4, 5]. In agriculture, a proteomic approach was used to investigate population growth and the effect of global climate changes on crop production [6]. In food technology, proteomics is utilized for characterization and standardization of raw materials, process development, and detection of batch-to-batch variations and quality control of the final product, in particular to food safety in terms of microbial content and the use of genetically modified foods [7]. The study of interactions between microbial pathogens and their hosts is called “infectomics” and comprises a growing area of interest in proteomics [8].

A protein may exist in multiple forms within a cell or cell type. These protein isoforms derive from transcriptional, post-transcriptional, translational, post-translational, regulatory, and degrading and preserving processes that affect protein structure, localization, function, and turnover. The field has thus evolved to include a variety of methods for separation of complex protein samples followed by identification using mass spectrometry. It is inherently a systems science that considers not only protein abundances in a cell but also the interplay of proteins, protein complexes, signaling pathways, and networks. To address the relevant challenges, we categorize the analytical tools into three types: (1) basic traditional statistical analysis, (2) machine learning approaches, and (3) assignment of functional and biological information to describe and understand protein interaction networks.

Traditional statistics is used as a critical first pass to identify the “low-hanging fruit” in the dataset. Methods such as *t* test and its nonparametric equivalent, the Wilcoxon test, univariate, or analysis of variance (ANOVA) are applied to identify the significant proteins. Due to inherent variability, statistics alone is often insufficient to discover most of the biologically relevant information in a proteomic dataset but is an important first step of every analysis. For the purposes of this review, we focus mainly on approaches that are more specific to proteomic and other “omic” data. But statistically significant results are very useful as seed data or bait in the machine learning approaches.

Machine learning classification complements traditional statistics as it allows for consideration of many variables at once and also removes much of the researcher bias. Dataset complexity is reduced as correlations, and trends are identified that may not withstand statistical scrutiny or may be undetectable using traditional statistics, e.g., clustering using iterative subsampling. Machine learning also bypasses researcher bias by revealing patterns within the data that may not relate to the original hypothesis or that relate in an unanticipated manner. The researcher is then able to examine the clustering or classification results for new biological features that were not initially predicted. Thus, in addition to being potentially inconsistent with the hypotheses of any particular researcher, machine learning and network tools enable hypothesis generation as they uncover the real biology of the system in question. Swan et al. [9] discussed the benefit of machine learning methods for application to proteomic data and show that machine learning methods give an overall view of data and also offer a large potential for identifying relevant information within data.

Pathway analysis following statistical analysis and classification and clustering can help organize a long list of proteins onto a short list of pathway knowledge maps, easing interpretation of the molecular mechanisms underlying altered proteins or their expressions [10].

Here we primarily review tools for machine learning and clustering of omic data. The machine learning section of this review will introduce the concept of supervised and unsupervised classification for seven types of machine learners: principal component analysis (PCA), independent component analysis (ICA), K-means, hierarchical clustering, partial least square (PLS), random forests (RF), and support vector machines (SVM). These methods are also summarized and compared in Table 1, which provides an overview of different machine learning and clustering tools and how to select a method most likely to be effective for a specific dataset. We include a brief discussion of experimental design and feature selection, i.e., the selection of significant attributes for reduction of datasets, with the aim to increase the accuracy of classification models that are applied to the selected features. The machine learning and clustering section is followed by a brief summary of tools for analysis of longitudinal (time series) data. Next, we discuss tools that can achieve automated learning of pathway modules and features and those that help perform integrated network visual analytics. Finally, we provide information for public repository of proteomics data.

**Table 1:** Summary and comparison of classification and clustering methods

	Classification					Clustering	
	PCA	ICA	RF	PLS	SVM	K-means	Hierarchical
What does it do?	Separates features into groups based on commonality and reports the weight of each component’s contribution to the separation	Separates features into groups by eliminating correlation and reports the weight of each component’s contribution to the separation	Separates features into groups based on commonality; identifies important predictors	Separates features into groups based on maximal covariation and reports the contribution of each variable	Uses a user-specified kernel function to quantify the similarity between any pair of instances and create a classifier	Separates features into clusters of similar expression patterns	Clusters treatment groups, features, or samples into a dendrogram
By what mechanism?	Orthogonal transformation; transfers a set of correlated variables into a new set of uncorrelated variables	Nonlinear, non-orthogonal transformation; standardizes each variable to a unit variance and zero mean	Uses an ensemble classifier that consists of many decision trees	Multivariate regression	Finds a decision boundary maximizing the distance to nearby positive and negative examples	Compares and groups magnitudes of changes in the means into K clusters where K is defined by the user	Compares all samples using either agglomerative or divisive algorithms with distance and linkage functions
Strengths	Unsupervised, nonparametric, useful for reducing dimensions before using supervision	Works well when other approaches do not because data are not normally distributed	Robust to outliers and noise; gives useful internal estimates of error; resistant to overtraining	Diverse experiments that have the same features are made comparable; variables can outnumber features	Robust to outliers, gives useful internal estimates of error, can exploit knowledge of the domain if using appropriate kernel functions	Easily visualized and intuitive; greatly reduces complexity; performs well when distance information between data points is important to clustering	Unsupervised; easily visualized and intuitive
Weaknesses	Number of features must exceed number of treatment groups	Features are assumed to be independent when they actually may be dependent	Does not allow missing data (requires imputation to replace missing values)	Fails to deal with data containing outliers	Selection of an inappropriate kernel yields poor results	Sensitive to initial conditions and specified number of clusters (K)	Does not provide feature contributions; not iterative, therefore, sensitive to cluster distance measures and noise/outliers
More information			Performance depends on number of trees and varies among experiments	Supervised; requires training and testing; groups pre-defined	Supervised; requires training and testing; many good kernel functions have been described, e.g., based on structural alignment	Tools are available to determine the optimal cluster count (K)	User does not define the number of clusters
Sample size/data characteristics	Unlimited sample size, data normally distributed	Unlimited sample size; data non-normally distributed	Performs well on small sample size and is resistant to over-fitting	Unlimited sample size; sensitive to outliers	Performs well on small sample size and resistant to over-fitting	Performs best with a limited dataset, i.e., ~20 to 300 features	Performs best with limited dataset, i.e., ~20 to 300 features or samples

## Experimental design

Although the purpose of this review is to discuss tools that are useful for data analysis after completion of a proteomic experiment, we want to recognize the essential nature of thoughtful upfront experimental design. Sample groups should be as large and reproducible as possible, representing a consistent proteomic phenotype in the harvested sample for a particular sample group. Even when the researcher is not establishing a study in a prospective manner, samples and sample groups should be chosen to reflect this insofar as is possible for the researcher. For example, if the experimental purpose is to find changes in the mouse hypothalamus with respect to circadian rhythm, the surgeries should—ideally—be performed by the same researcher at precise times of the day until a minimum of five or six samples, preferably more, are collected for every treatment group in question. The power of the experiment increases with each additional sample. Treatment groups should ideally be similar in size. Consistent collection, storage, and sample handling during the experiment will greatly increase chances of high-quality omic data. Furthermore, reduction of a sample to fewer or a specific cell type will increase the quality of proteomic or RNA data. Gene expression is a cell-type-specific phenomenon so that, in order to increase the signal-to-noise ratio for a gene expression study, the experimental design should consider tissue and sample complexity. A protein extract from liver, for example, primarily comprises hepatocyte proteins, whereas the brain contains cells that express hugely variable mRNA and protein signatures. We encourage the researcher to plan carefully regarding experimental design, as this investment will yield greatly improved resulting data. For review of experimental design, see [11, 12].

## Guidelines for analyzing a large dataset

The following guidelines are listed as sequential steps, but they are meant to be more of a frame for thought rather than rigid steps in a series. For example, steps one and three may overlap and provide answers to the main questions of the experiment. Step two may obviate the need to perform extensive machine learning. Our hope is to relieve the distress of inheriting or creating an enormous mass of data that seems impenetrable.

### Step one: Observe your data, quality control

Observe your data by creating plots and descriptive statistics to assess data distribution, overall variation, and variability within each treatment group. Compare means and variability from those means. Look for any anomalies that could cause a problem in the analysis. Plotting the data is effectively the first unsupervised clustering step. How do the data cluster? Are the data normally distributed? Most parametric statistical approaches assume normality, so if data are not normally distributed, they may need to be transformed or analyzed using nonparametric methods. Curves, scatter plots, and boxplots are useful for observing comparability of different groups or whether two different datasets can be combined. Is there a batch effect? If so, the data must be normalized or corrected for this effect. If using unsupervised approaches such as hierarchical clustering or principal component analysis, do the subjects partition according to predicted treatment groups? Correlation plots can be used to compare treatment groups. Are the correlations as expected?

### Step two: Traditional statistics

Groups identified by the researcher either during experimental design or during the data observation step can be compared here using Student’s *t* test, analysis of variance (ANOVA), and their nonparametric equivalents such as Kruskal-Wallis, in addition to regression modeling and other tests of traditional statistics. Many tests done simultaneously should be corrected using a multiple test correction such as the Benjamini-Hochberg correction algorithm [13]. If these tests yield an abundance of significant data, the machine learning methods of step three can be used to reduce dimensionality. These lists of significant features can be used directly for pathway analysis. Or alternately, these significant features can be used as a seed or paradigm for training the supervised machine learning methods in step three to retrieve interesting data that were not found to be significant by traditional statistical methods.

For example, suppose we identify 100 significant features (proteins, transcripts, etc.) after multiple test correction. These 100 can be tested internally for correlation, for pattern recurrence, and for pathway analysis (DAVID, GO, Ingenuity, etc., Table 2). Suppose we used K-means to look for ten patterns, and one of the ten patterns happens to contain five features whose expression profiles appear to match what we know about their biology based on previous experiments or established literature. This is the step we might call “kicking the tires” of this dataset. If gene expression for a few proteins or transcripts follows known patterns, the entire dataset becomes more credible; other significant data can thus be relied upon as informative for further analysis and for interrogating the rest of the data.

**Table 2:** Summary of functional and network tools

Name	Description	Link	References	Function
KEGG	Kyoto Encyclopedia of Genes and Genomes	http://www.genome.jp/kegg/	Kanehisa and Goto (2000) [76]	Pathway
DAVID	The Database for Annotation, Visualization and Integrated Discovery	http://david.abcc.ncifcrf.gov/	Dennis et al. (2003) [96]	Pathway and functional annotation using GO
PID	Pathway Interaction Database	http://pid.nci.nih.gov/	Schaefer et al. (2009) [97]	Pathway interaction
IPA	Ingenuity Pathway Analysis	http://www.ingenuity.com/		Pathway and functional annotation
Cytoscape	An open source platform for complex network analysis and visualization	http://www.cytoscape.org/	Shannon et al. (2003) [98]	Network visualization
HAPPI	Human Annotated and Predicted Protein Interaction Database	http://bio.informatics.iupui.edu/HAPPI	Chen et al. (2009) [99]	Protein interaction
GSEA	Gene Set Enrichment Analysis	http://www.broadinstitute.org/gsea/	Subramanian et al. (2005) [77]	Pathway analysis and functional annotation
Reactome	Curated database of pathways and reactions (pathway steps)	http://www.reactome.org/	Matthews et al. (2009) [100]	Pathway
BioCarta	Pathway database	http://www.biocarta.com/	Nishimura (2001) [101]	Pathway
HPD	Integrated Human Pathway Database	http://discovery.informatics.iupui.edu/HPD/	Chowbina et al. (2009) [102]	Pathway
PAGED	Pathway and Gene Enrichment Database	http://omictools.com/paged-s3492.html	Huang et al. (2012) [103]	Pathway, functional annotation
HPRDB	Human Protein Reference Database	http://www.hprd.org/	Keshava Prasad, T. S. et al. (2009) [104]	Annotation
DrugBank	Drug Bank	http://www.drugbank.ca/		Combines drug data with drug target
CPDB	Consensus Path DB	http://consensuspathdb.org/	Kamburov, A. et al. (2013) [105]	Interaction networks (protein-protein, genetic, metabolic, signaling, gene regulatory, and drug-target)
BINGO	Biological Network Gene Ontology Tool	http://www.psb.ugent.be/cbd/papers/BiNGO/Home.html	Maere S, Heymans K, and Kuiper M (2005) [106]	Biological network gene ontology
GATHER	Gene Annotation Tool to Help Explain Relationships	http://gather.genome.duke.edu	Chang JT, and Nevins JR. (2006) [84]	Gene annotation tool

From these lists, one can transition directly to pathway analysis (step four), or these data can be used for classification of the rest of the dataset using machine learning methods.

### Step three: Dimension reduction with machine learning

The “curse of dimensionality” is inherent to large datasets. At the beginning of any large dataset analysis, the dimension count and the feature count are the same. The purpose of machine learning is to reduce the dimensions such that multiple features (or data points) are contained within a single dimension so that a dataset with 5000 features may contain 500 groups of ten features each where those ten features have something in common as determined by the classifier such as PCA, RF, and K-means. Thus, machine learning allows the data to partition according to the biology of the experiment, and it allows the researcher to better comprehend the data and the potential biological processes that drive the experimental question.

Many machine learning tools are available including Weka [14], Scikit-learn (Machine Learning in Python) [15], and SHOGUN [16]. R has an enormous number of machine learning algorithms with advanced implementations as well that were written by the developers of the algorithm [17].

If performed independently, machine learning and traditional statistics ought to reveal the same results in the data. They confirm each other. As stated in Table 1, different tools for machine learning are appropriate for different datasets. The observation of data in step one will help the researcher to identify which statistics and machine learning approaches might prove to be most effective in partitioning the data in question. For example, if data are not normally distributed and transformation of the data is not desirable, one should start by using nonparametric statistical analyses and independent component analysis.

### Step four: Pathway analysis

Genes and features of interest are entered into pathway analysis software and tools, which are rapidly increasing in sophistication. Still, we have found that computational tools for pathway analysis should always be supplemented with individual manual research into relevant literature and textbook information for real biological insights. Only when the individual researcher or team is able to absorb the biological implications of the new data will the true understanding take place. The computational tools enable new connections to be established, but the biological story still requires concept synthesis on the part of the researcher.

## Machine learning and clustering methods

It is reasonable to assume on biological grounds that the proteins present in the proteomic profile are not fully independent of each other in vivo. For this reason, a multivariate approach to analysis is preferred because it can address the correlations among variables. Dimension reduction methods project a large number of genes or proteins onto a smaller and more manageable number of features. The art of machine learning starts with the design of appropriate data representations, and better performance is often achieved using features derived from the original input and experimental design of the researcher. Building a feature representation is an opportunity to incorporate domain knowledge into the data and can be very application-specific. Nonetheless, there are a number of generic feature construction methods, including the following: clustering, basic linear transforms of the input variables (PCA/ICA/PLS), more sophisticated linear transforms like spectral transforms (Fourier, Hadamard), convolutions and kernels, and applying simple functions to subsets of variables. Among these techniques, some of the most important approaches include (i) dimensionality reduction, (ii) feature selection, and (iii) feature extraction.

There are many benefits regarding the dimensionality reduction when the datasets have a large number of features. Machine learning algorithms work best when the dimensionality is lower (curse of dimensionality). Additionally, the reduction of dimensionality can eliminate irrelevant features, reduce noise, and produce more robust learning models due to the involvement of fewer features. In general, the dimensionality reduction by selecting new features which are a subset of the old ones is known as feature selection. Three main approaches exist for feature selection, namely the following: embedded, filter, and wrapper approaches [18]. In the case of feature extraction, a new set of features can be created from the initial set that captures all the significant information in a dataset. The creation of new sets of features allows for gathering the described benefits of dimensionality reduction.

Sometimes classifications or clustering decisions are susceptible to high bias (under-fitting) or high variance and low bias (over-fitting). If there is under-fitting that results in a high error rate in both training and test, it might help to (1) add more features, (2) use a more sophisticated model, or (3) employ fewer samples. If the dataset has a high variance and low bias (over-fitting) that results in a low error rate in training but high error rate in the test case, it might help to (1) use fewer features or (2) use more training samples. Over-fitting is usually a more common problem in classification than under-fitting. Over-fitting the data causes the model to fit the noise rather than the actual underlying behavior.

The application of different feature selection techniques usually produces different predictive feature lists, presumably because each method captures different features from the data or the small number of samples.

Classification methods have been used extensively for visualization and classification of high-throughput data. These algorithms group objects based on a similarity metric that is computed for features. There are several issues that can affect the outcome of the methods, including (1) a large number of features, (2) mean of the groups, (3) variance and (4) correlation among groups, (5) distribution of the data, and (6) outliers. Thus, exploiting the hidden structure within a dataset is critical for improving classification selection and accuracy and speed of prediction systems. No free lunch (NFL) theorems previously showed that any two optimization algorithms are equivalent when their performance is averaged across all possible problems [19, 20]. Here we emphasize the importance of the hidden structure of the data in order to achieve superior performance of learning systems.

Supervised machine learning involves training a model based on data samples that have known class labels associated with them. This is in contrast with unsupervised classification, or clustering, where no samples have associated class labels, and instead, samples with similar attribute profiles are grouped together.

Each of the supervised classification methods described can make errors, either by incorrectly identifying an instance as a member of a class (a “false positive”) or by incorrectly failing to identify an instance as a member of a class (a “false negative”). The rates of both types of errors can be estimated; the proportion of false positive results is reported using *specificity* and the proportion of false negatives using *sensitivity*. There is often a trade-off between these types of errors; increases in specificity (fewer false positives) often lead to decreases in sensitivity (more false negatives) and vice versa. Some classification methods always treat these types of errors as equally important, but others allow the user to set an explicit trade-off ratio, e.g., telling the classifier that sensitivity is twice as important as specificity or vice versa. Methods that have adjustable sensitivity/specificity trade-offs are noted in Table 1. There are no “one size fits all” tests in classification or clustering methods, and different datasets can make errors which are specific to that dataset (i.e., the no free lunch theorem).

### Unsupervised classification and clustering

#### Principal component

The principal component analysis (PCA) [21] is a mathematical procedure that transforms a number of possibly correlated variables into a smaller number of uncorrelated variables, which are then ordered by reducing variability. These variables are called principal components. The first principal component accounts for as much of the variability in the data as possible, and each succeeding component accounts for as much of the remaining variability as possible. PCA is an unsupervised analysis tool since samples are classified without including disease status in the training algorithm and best if the variables are standardized, and in most of the implementation, this is done by default. PCA is not only useful as a visualization tool [22]. It also helps to detect outliers and perform quality control. PCA has been widely used in analysis of high-throughput data including proteomic data, e.g., [23–25].

#### Independent component

Independent component analysis (ICA) [26] is a method for finding underlying factors or components from multidimensional data. ICA is also known as blind signal separation (BSS). PCA and ICA have very different goals, and naturally, they may give quite different results. PCA finds directions of maximal variance (using second-order statistics) while ICA finds directions that maximize independence (using higher order statistics) [27]. ICA maximizes non-Gaussianity and makes the assumption of combinatorial linearity of components, satisfied by removing the correlated data. In contrast to PCA, ICA analysis seeks not a set of orthogonal components but a set of independent components. Two components are independent if any knowledge about one implies nothing about the other, such that independent components (IC) represent different non-overlapping information. Since the number of components can be very high, it is relatively easy for the ICA estimation to over-fit the data.

Safavi et al. used ICA to separate groups of proteins that may be differentially expressed across treatment groups [28]. They also showed that the univariate ANOVA technique with false discovery rate (FDR) correction is very sensitive to the FDR-derived *p* value, whereas ICA is able to identify and separate differential expression into the correct factors without any *p* value threshold. Other studies have applied ICA to MS data and have shown that ICA represents a powerful unsupervised technique [29, 30].

#### K-means

K-means [31, 32] is a popular partitioning method due to its ease of programming, allowing a good trade-off between achieved performance and computational complexity. It performs well when the distance information between data points is important to the clustering. K-means requires the analyst to specify the number of clusters to extract, and there are tools available to determine the appropriate number of clusters [33]. Although this is a widely used technique, it suffers from several drawbacks: K-means does not scale well with high dimensional datasets and is prone to local minima problems. It is sensitive to initial conditions, does not remove undesirable features for clustering, and it is best but even then it is prone to local maxima. In spite of the weaknesses, with thoughtful application, the K-means algorithm is very useful in analysis of proteomics data due to its simple algorithmic assumptions and intuitively clear and interpretable visualization [34, 35].

#### Hierarchical clustering

Hierarchical clustering outputs a dendrogram tree representation of the data. Leaves are the input patterns and non-leaf nodes represent a hierarchy of groupings. This method comes in two flavors: agglomerative and divisive. Agglomerative algorithms work from the bottom up, with each pattern in a separate cluster. Clusters are then iteratively merged according to some criterion. Conversely, divisive algorithms start from the whole dataset in a single cluster and work top down by iteratively dividing each cluster into two components until all clusters are singletons. Hierarchical clustering suffers from the disadvantage of any merging/division decision being irreversible and any errors being dragged through the rest of the hierarchy (in another word, established mergers cannot be undone). Thus, hierarchical clustering analysis and principal component analysis can be used to identify subgroups on the basis of similarities between the proteins’ expression profile. Hierarchical clustering methodologies commonly used in transcriptomic studies have also been performed on proteomic data [36, 37]. The different methods will shed light on different aspects of the data [38, 39].

### Supervised classification

#### Partial least squares

Partial least squares (PLS) [40] is a method of dimensionality reduction that maximizes the covariance between groups. PLS constructs a set of orthogonal components that maximize the sample covariance between the response and the linear combination of the predictor variables. It generalizes and combines the features of PCA and multilinear regression [41, 42]. Through maximizing the covariance of dependent and independent variables, PLS searches for the components that capture the majority of the information contained in independent variables as well as in the relations between dependent and independent variables. PLS regression is particularly useful when users have a very large set of predictors that are highly collinear. In case of over-fitting, the PLS will (1) reduce the predictors to a smaller set of uncorrelated components—these components are mapped in a new space—and (2) perform least squares regression on the new set of components. Although PLS regression was not originally designed for classification and discrimination problems, it has often been used for this purpose [23, 25, 43–49].

#### Random forests

Random forests (RF) [50] are another classifier method that consists of many decision trees and can be either supervised or unsupervised. It is a popular method that has gained recognition for its ability to construct robust classifiers and select discriminant variables in proteomics [34, 35, 51–54].

RF is an extension to bagging and uses *de-correlated* trees; it is capable of minimizing the number of selected features. For a given decision tree, a subset of samples is selected to build the tree; the remaining samples are predicted from this tree. Bagging (bootstrap aggregating) can be used as an ensemble method [55]. To see which variables contribute the most to the separation, “importance” measures are computed, e.g., the “mean decrease accuracy” and the Gini index [50].

Principal component analyses are used for dimension reduction, but the reduction is valid only when the number of components (i.e., subjects in a study) is less than the number of features (i.e., measured entities in the experiment). In contrast, random forests can be used when the number of features (metabolites, genes, or proteins) is smaller than the number of subjects. A random forest tends to be resistant to over-fitting and also not very sensitive to outliers. A random forest does not handle missing data, and missing values either need to be eliminated or imputation of missing data is needed.

#### Support vector machine

Support vector machine (SVM) [56] is a supervised learning method that constructs a hyperplane or set of hyperplanes in a high-dimension or infinite dimensional space. A good separation is achieved when the hyperplane has the largest distance to the nearest training data point of any class (the so-called functional margin).

SVM can be applied to different data types by designing the kernel function for such data; selection of a specific kernel and parameters is usually a trial and error process. A kernel function is one that corresponds to an inner product in some expanded feature space. Kernel methods are a kernel class of algorithms for pattern analysis. Since SVM is using regularization, it is highly resistant to over-fitting, even in cases where the number of attributes is greater than the number of observations. In practice, this depends on the careful choice of a C and kernel parameter. A C parameter is an optimization or regularization parameter which is chosen by the user to allow the SVM to best classify the training set. For larger C, the optimization will choose a smaller margin hyperplane if that does a better job of getting all the training points classified correctly. For a very small value of C, this will cause the optimizer to look for a larger margin-separating hyperplane even if that hyperplane misclassifies more points. SVM has been used in various fields to identify biomarkers including proteomics datasets [57–60].

### Longitudinal or time-series data

Several software tools are available that specifically address the problems associated with time-series data. TimeClust is a stand-alone tool which is available for different platforms and allows the clustering of gene expression data collected over time with distance-based, model-based, and template-based methods [61]. There are also several other packages available in R such as maSigPro [62], timecourse [63], BAT [64], betr [65], fpca [66], timeclip [67], rnits [68], and STEM [69].

Python probabilistic graphical query language (pGQL) [70] allows its user to interactively define linear HMM queries on time-course data using rectangular graphical widgets called probabilistic time boxes. The analysis is fully interactive, and the graphical display shows the time courses along with the graphical query. In JAVA, PESTS [71] and OPTricluster [72] both of which are stand-alone with a GUI interface are useful for the clustering of short time-series data in MATLAB. DynamiteC is a dynamic modeling and clustering algorithm which interleaves clustering time-course gene expression data with estimation of dynamic models of their response by biologically meaningful parameters [73].

### Pathway analysis

After statistical and/or machine learning analysis, the next challenge is how to extract functional and biological information from a long list of proteins identified or discovered from high-throughput proteomic experiments. In order to provide biological insights into the underlying molecular mechanisms of different conditions [10] or changes involved during the progression of disease as well as identification of potential drug targets [74–76], pathway and network analysis techniques can help to address the challenges of interpretation. We categorize these tools into three types: (1) tools with basic functional information (e.g., GO category analysis), (2) tools with rich functional information and topological features (e.g., GSEA [77], IPA [78]), and (3) tools with topological features (e.g., Cytoscape [79]).

For pathway analysis, we refer to data analysis that aims to identify activated pathways or pathway modules from functional proteomic data. For network analysis, we refer to data analysis that builds, overlays, visualizes, and infers protein interaction networks from functional proteomics and other systems biology data. It is at this stage that metabolomic and proteomic data intersect to reveal active biological processes in a particular system.

Pathway Commons [80] is publicly available and has pathway information for multiple organisms. Pathways include biochemical interactions, complex assembly, transport and catalysis events, physical interactions involving proteins, DNA, RNA, small molecules and complexes, genetic interactions, and co-expression relationships. HumanCyc plus Pathway Tools [81] provides another set of options. HumanCyc contains well-curated content on human metabolic pathways. The associated Pathway Tools software will let you paint gene expression, proteomics, or metabolomics data onto the HumanCyc pathway map, and Pathway Tools will also perform enrichment analysis. PathVisio [82] is a publicly available pathway editor and visualization and analysis software. 3Omics [83] is a web-based systems biology visualization tool for integrating human transcriptomic, proteomic, and metabolomic data. It covers and connects cascades from transcripts, proteins, and metabolites and provides five commonly used analyses including correlation network, co-expression, phenotype generation, KEGG/HumanCyc pathway enrichment, and GO enrichment. For these tools, the user uploads transcriptome and proteome expression data. The metabolome is inferred using KEGG Pathway. 3Omics derives the relationship between the proteome and the metabolome from the literature.

GSEA [77] enables molecular-signature-based statistical significance testing, which integrates protein functional category information effectively with statistical testing of functional genomics or proteomics results. GATHER [84] is a functional enrichment tool (for KEGG pathways) along with several other categories which provides information for a list of genes/proteins in the context of genes, GO terms, predicted miRNAs, pathways, or diseases. The Protein ANalysis THrough Evolutionary Relationships (PANTHER) [85] classification system is designed to classify proteins (and their genes) to support high-throughput analysis. It combines human curation with gene ontology and utilizes other sources for high-level analysis of protein lists.

A number of visualization tools and plug-ins are available for Cytoscape [79] which can be used for biological network construction.

Ultimately, future tools must support elucidation of complex molecular mechanisms suggested from multiscale network data and molecular signature data. However, there are still significant challenges in designing next-generation network/pathway analysis tools. Network analysis and pathway analysis have been extensively applied to proteomic datasets, e.g., [75, 86, 87]. Some of the pathway and network analysis tools that have become available in the last decade are listed in Table 2. Although the content of most of these tools is based on knowledge and is freely available, a user might not be able to reproduce the same result using a different selection of tools. These tools integrate information from different sources; they obtain pathway information from the literature and by computational prediction.

### Proteomics data repositories

There has been great progress in the last few years in making raw proteomic data publicly available, which provides a considerable value to the community. Currently, several repositories compile proteomic data. The PRoteomics IDEntifications (PRIDE) [88] database at the EBI is a public repository that includes protein and peptide identifications, post-translational modifications, and supporting spectral evidence. The PeptideAtlas database [89] from ISB’s Proteome Center accepts only the raw output of mass spectrometers, and all raw data are processed through a uniform pipeline of search software plus validation with the Trans-Proteomic Pipeline (TPP) [90]. The results of this processing are coalesced and made available to the community through a series of builds for different organisms or sample types.

The *Mass* spectrometry *I*nteractive *V*irtual *E*nvironment (MassIVE) is a community resource developed by the NIH-funded Center for Computational Mass Spectrometry to promote the global, free exchange of mass spectrometry data [91]. The MassIVE can be run with UCSD proteomics [92]. Chorus is a simple web application for storing, sharing, visualizing, and analyzing spectrometry files [93]. A user can upload experiment files along with the metadata, analyze them, and also make them available to collaborators. The Global Proteome Machine Database (GPMDB) collects spectra and identifications that have been uploaded by researchers to a GPM analysis engine and presents the summarized results back to the community [94].

To make the process of data submission easier for the user, the ProteomeXchange consortium is set up to provide a single point of submission to proteomics repositories [95]. Once the data are submitted to the ProteomeXchange entry point, they can be automatically distributed to all other repositories (PRIDE, MassIVE, and PeptideAtlas).

## Discussion and conclusion

Machine learning and clustering approaches have been applied to proteomic and mass spectrometric data from many different biological disciplines in order to identify biomarkers for normal phenotypic characterization [38] and for diagnosis, prognosis, and treatment of specific disease [48, 57]. The bioinformatics tools that are currently available for omic data analysis span a large panel of very diverse applications ranging from simple tools to sophisticated software for large-scale analysis. Technical advances and growing interest in the field have given rise to a great number of specialized tools and software to derive biologically meaningful information. These computational approaches assist in generating hypotheses to be tested in orthogonal experiments.

Machine learning and its methods have increasingly gained attention in bioinformatics research. With the availability of different types of classification methods, it is common for researchers to apply these tools to classify and mine their data. But one should keep in mind that no matter how sophisticated the bioinformatics tools, the quality of the results they produce is directly dependent on the quality of input data they are given. In addition, new experimental methods are likely to require newly adapted bioinformatics tools as mass spectrometers become more powerful and as novel experimental design results in more complex datasets. One area of rapidly expanding complexity is at the integration of the fronts of metabolomic and proteomic data. Each software tool has some advantage and disadvantage, so it benefits the user to employ a combination of tools to examine one dataset rather than a single software tool. Each dataset contains its own quirks, positive and negative, and it is up to the end users and analysts to decide the most effective approach for assessing the biology that is taking place within their experiment.
